# Implications of Aging in Place in the Context of the Residential Environment: Bibliometric Analysis and Literature Review

**DOI:** 10.3390/ijerph20206905

**Published:** 2023-10-10

**Authors:** Eugene Seo, Sanghee Lee

**Affiliations:** Department of Architecture, Kwangwoon University, Seoul 01897, Republic of Korea; spes912@kw.ac.kr

**Keywords:** aging in place, aging in community, aging at home, residential environments, bibliometric analysis

## Abstract

The residential environment’s impact on aging in place is a multidisciplinary field that draws from architecture, urban planning, gerontology, psychology, and sociology. This multidisciplinary nature makes it challenging to comprehensively understand the field and identify the connections and interactions among disciplines. A bibliometric analysis is crucial for exploring the field’s intellectual structure, identifying interdisciplinary collaborations, and tracking the knowledge flow across disciplines and will facilitate cross-disciplinary dialogue, foster collaboration, and encourage research that integrates diverse perspectives. This study reviewed the literature on aging in place in the context of a residential environment, which required adapting theories and methodologies. It analyzed a dataset of 1500 publications retrieved from the Web of Science, applied performance analysis techniques, and utilized VOSviewer to visualize the intellectual structure and evolving research themes. The results emphasize the increasing strength of academic interest and the growing diversity of fields related to the topic. The findings are discussed in terms of productivity, collaboration, and research themes from the past to the future. The results provide a roadmap for researchers, policymakers, and practitioners worldwide who focus on aging in place and acknowledge the importance of considering the physical, social, and cultural aspects of an older adult’s living environment.

## 1. Introduction

The elderly population is rapidly growing due to advances in medical technology and the aging of the baby boomer generation. According to data from World Population Prospects [1], by 2050, one in six people in the world will be over the age of 65 (16%), up from Additionally, it is anticipated that by 2050, individuals aged 65 or above will comprise one quarter of the population in Europe and North America. As of 2018, those aged 65 or above surpassed the number of children under five for the first time in recorded history [1]. Forecasts suggest the population of those aged 80 or above will experience a threefold increase, escalating from 143 million in 2019 to 426 million in 2050 [2].

This significant increase in the older age groups will inevitably result in increased chronic morbidity and functional disabilities. Changes in lifestyles, needs, and expectations due to demographic aging will continue to substantially evolve and particularly have implications for how society approaches the aging process and the environment in later life [3]. Western societies have been reassessing residential systems for older people, recognizing this issue as one of the major challenges of our time [4].

Housing and community environments (i.e., the spaces where daily life occurs) have been identified both in policy and by older people themselves as preferred settings for care and support, as they allow for autonomy and privacy compared to institutional settings [5,6]. Many countries have adopted initiatives promoting home care and community-based elderly care for both economic and well-being reasons [7]. The outcome is clear: most older people want to live in their own homes as long as possible [5,8,9] even though their homes are often unsuitable for their aging years [10]. Scientific literature and policy programs examine this preference through the concept of “aging in place” [3].

The accelerated aging observed in numerous Western societies has urged policymakers and experts to formulate concepts, initiatives, and services designed to accommodate the multifaceted and varied needs of the aging population, especially those who are fragile, suffer from chronic illnesses, or have functional disabilities. The concept of aging in place has emerged as a principal and orienting approach to respond to and fulfill the requirements of the elderly. The aging in place concept has expanded to incorporate interdisciplinary studies beyond the realm of gerontology and administration to understand the relationship between seniors and their environments [11]. Despite the increasing number of studies on aging in place, several associated challenges still need to be addressed regarding a holistic approach, one that extends beyond the limited focus on environmental factors such as physical accessibility and functional adaptations [12]. Although research on aging in place is thriving due to the aging phenomenon, only a limited number of studies have considered environmental factors. According to Clarke and Gallagher (2013), the research has predominantly focused on individuals rather than environmental considerations, as mobility is the most prevalent form of disability that today’s older adults face [13]. Most research conducted on older people’s residential environments has focused on barrier-free renovations, such as physical accessibility and functional adaptations, to remove mobility barriers and reduce the risk of falls [14]. 

However, aging in place is a semantically broad concept [5,15], and an effective practical response requires a comprehensive understanding of seniors’ living environments. In light of this, our aim was to understand how aging in place has been conceptualized in relation to residential environments and explore how it has been addressed in the research literature. We conducted a scoping review to trace the evolution of the definitions of aging in place over time and across disciplines to understand the parameters of elderly living. In order to identify the unique contributions of this paper and the research gaps in the existing literature, we conducted a search on the Web of Science using the keywords “aging in place” and “environment” and looked for papers involving systematic analysis, bibliometric analysis, or PRISMA analysis. A total of 14 papers were retrieved, of which 5 were from a technology perspective. The remaining papers were related to the psychological well-being of the elderly, social support, and elderly home care. We have confirmed that existing studies are lacking in providing a holistic approach to the concept of aging in place and the residential environment, thereby identifying the research gap this paper aims to fill.

This research did not limit the scope of aging in place to the architectural domain but also includes multidisciplinary findings. Furthermore, it explored scientific productivity and the intellectual collaborations of publications and researchers in the field of aging in place. Specifically, this study focuses on key aging in place concepts and presents a scoping review of the theoretical and methodological trends.

## 2. Materials and Methods

This study employs bibliometric methodology, utilizing quantitative techniques to analyze bibliometric data. We accessed scientific databases like the Web of Science to obtain extensive bibliometric data on the research topic. The analysis was facilitated by the bibliometric software VOSviewer, which enabled a comprehensive examination of research trends across various disciplines, including multidisciplinary studies [16,17,18], finance [19], urban studies [20,21], and more. This approach allowed us to explore the intellectual structure and prolific aspects of the research topic of aging in place. The bibliometric study was conducted to include the Preferred Reporting Items for Systematic Reviews for Scoping Reviews (PRISMA-ScR) [22] using the following steps:

Step 1 Define the objective and scope of the study;
Step 2 Select the appropriate techniques for bibliometric analysis;
Step 3 Collect the necessary data for bibliometric analysis;
Step 4 Perform the bibliometric analysis, including performance analysis and science mapping;
Step 5 Present the findings and discuss their implications for future research.

By following these steps, our goal was to use a bibliometric approach to provide insights into the evolving research trends of aging in place across various disciplines.

### 2.1. Define the Aim and Scope of the Research

In the literature, there is often confusion surrounding the use of terms such as “aging in place” and “aging in community”, which are frequently used interchangeably. However, this study specifically focuses on the environmental field as it applies to the residence and community dimensions of aging in place. This bibliometric analysis aims to provide an overview of how the term “aging in place” is used in the literature and identify the common disciplines that contribute to developing related theoretical concepts and methodologies.

Additionally, this bibliometric analysis provides theoretical and methodological references for aging in place in the context of elderly living. Consequently, it can help researchers understand the gaps between the disciplines and generate new ideas for successfully implementing aging in place initiatives. Therefore, this study aims to inform the development of future research so researchers can apply the residential-environment development and elderly-living model approach to aging in place.

The bibliometric analysis addresses the following research questions:

RQ1. Which research area is leading in the field of aging in place in the context of residential environments?

RQ2. What methodologies have been applied in aging in place research? 

RQ3. How are the theory and methodology of aging in place research evolving?

RQ4: What challenges and implications does aging in place have for residential environments?

### 2.2. Framework of the Research

Table 1 presents the selection and analysis process followed in this study. The data collection process consisted of two main steps. In the first step, the titles, abstracts, and keywords of primary studies were examined to gather relevant articles for further analysis. In the second step, a bibliometric approach was applied to conduct a descriptive and quantitative analysis of traditional literature reviews sourced from Web of Science databases. This analysis involved investigating various aspects, including the publication year and total number of citations, as well as identifying the most productive authors and countries and constructing a co-occurrence network of author keywords. These steps were undertaken to gain insights into the characteristics and trends of the literature within the study’s scope of aging in place.

### 2.3. Collecting Data

#### Literature Screening Process

The data collection process plays a crucial role in determining the validity and significance of research results. In this study, the research questions were designed to explore the bibliometric landscape using scientific databases, specifically the WoS Core Collection. A bibliometric analysis conducted on an electronic scientific database like the WoS permitted the addressing of the research questions identified and uncovered emerging trends and patterns in the field of aging in place. Utilizing reputable scientific databases ensured the data’s reliability and comprehensiveness [23]. 

The research method involved conducting a search in the WoS with the keywords “aging in place” and other terms related to the research topic, which were drawn from a preliminary literature review. The search terms employed were (“aging in place” OR “aging in community” OR “aging at home” OR “ageing in place” OR “ageing in community”) AND (housing OR home OR dwelling OR residence OR residential).

Following the PRISMA-ScR steps, the WoS search using these search terms identified a total of 2026 documents across all fields and document types. To refine the search results, terms appearing in journal names were excluded, and the remaining search terms were specifically applied to fields relevant to the research topic, including the title, abstract, author keywords, and Keywords Plus. This refinement resulted in a total of 1970 documents for further analysis.

This study’s focus was specifically on peer-reviewed journals to exclude review articles and minimize duplication of similar topics. Out of the selected articles, the document type “Selected articles” accounted for 1839 papers. Grey literature such as proceedings papers (176), meeting abstracts (97), editorial materials (22), book reviews (6), book chapters (1), corrections (1), and new items (1) were manually excluded, resulting in a total of 1535 papers. Additionally, 69 early-access articles were included in the analysis. English was chosen as the preferred language for the articles; thus, non-English articles were excluded, leaving 1506 papers for further analysis. Hidden review articles were also screened manually; those with the term “review” in the title were removed, resulting in 1500 papers. Among these, six articles were identified as scoping reviews. The publication years of the selected articles spanned the three decades from 1991 to 2023.

The criteria for article selection in this study were as follows:

1. Document type: only peer-reviewed articles;
2. Language: English;
3. Publication year range: 1991–2023.

The suggested sample size for bibliometric analysis is approximately 1000 papers [24], and the number of papers included in this study exceeded that recommendation. 

## 3. Bibliometric Analysis

Bibliometric analysis is broadly acknowledged as a rigorous approach for evaluating extensive amounts of scientific information, enabling researchers to identify evolving trends and scrutinize the intellectual framework of a specific field of study. In this research, techniques of both performance analysis and science mapping were utilized. By utilizing these techniques, the study aimed to provide a comprehensive understanding of the research landscape and identify influential research entities in the field of aging in place.

Performance analysis constitutes a comprehensive evaluation of pivotal research entities—including authors, academic institutions, nations, and scholarly journals—based on metrics such as the aggregate number of publications and citations received. Among the principal methodologies delineated in the literature by Donthu et al. [23], science mapping serves as a salient technique for investigating the interrelations among diverse research elements. Science mapping involves exploring the relationships between various research components. These components are categorized into five analysis techniques: citation analysis, co-citation analysis, bibliographic coupling, co-word analysis, and co-authorship analysis. These techniques have the following characteristics:

1. Citation analysis: This technique is employed to identify the relationships among the most influential publications in the aging in place research field. Analyzing citations can give researchers insights into the impact and influence of specific papers in the domain.
2. Co-citation analysis: Co-citation analysis helps uncover foundational thematic clusters and seminal publications by examining the relationships among cited publications based on their references. This analysis revealed common themes and influential works in the field of aging in place.
3. Bibliographic coupling: Bibliographic coupling focuses on identifying the periodical or current development of themes in the research field. This technique examined the relationships among citing publications, providing insights into evolving trends and developments within the field of aging in place.
4. Co-word analysis: Co-word analysis delves into the relationships among various topics in the aging in place research field. Analyzing the co-occurrence of words or terms in publications allows researchers to identify existing and potential relationships among topics, revealing the interconnections and trends in the field.
5. Co-authorship analysis: Co-authorship analysis scrutinizes the intellectual collaboration among authors and their affiliations and evaluates the impact of such collaborations on the research field’s development. This analysis aided in identifying influential authors, research networks, and patterns of collaboration in the study of aging in place.

### 3.1. Overview/Description of the Bibliometric Analysis Results to Identify Prolific Research (Performance Analysis)

The analysis conducted on the 1500 records listed in the WoS focused on publications related to “aging in place” or “aging in community”. Specifically, the analysis examined publications in which terms related to “housing” or “home” and terms such as “community”, “dwelling”, and “residence” appeared as the publication topic. By narrowing the scope down to these specific criteria, the analysis aimed to gain insights into the literature specifically related to the intersection of aging, housing, and community aspects in the context of aging in place or aging in community.

#### 3.1.1. Publications and Citations over the Years

**Figure 1:**
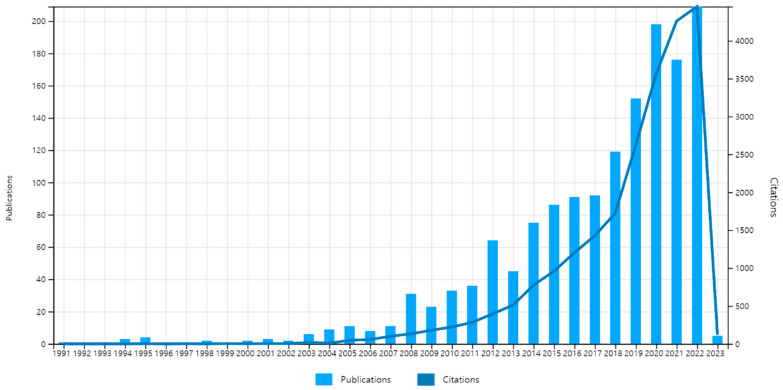
Number of citations and publications over time.

Figure 1 displays the annual changes in the number of relevant papers published from the 1990s to 2023, providing an overview of the research development trend in the field of aging in place. As Figure 1 shows, there were no more than five publications per year in this field until 2002. The data reveal steady growth in the number of relevant papers since 2003. Between 2000 and 2011, the number of publications related to aging in place experienced a moderate increase. However, from 2013 to 2021, there was a rapid increase in the number of publications and citations, indicating a substantial rise in attention to and research activity in this field. In particular, the data show a considerable increase in the number of publications and citations in 2012 and from 2018 to 2021. The notable increase in publications in 2012 can be attributed to an expansion of the number of journals involved in gerontology research. Specifically, the number of journals focusing on gerontology increased from 17 in 2011 to 33 in 2012. This expansion had a positive impact on the field, leading to an increase in the number of journals indexed in the Science Citation Index (SCI) from 10 to 18. Similarly, the number of indexed journals in the Social Science Citation Index (SSCI) also increased from 24 to 39 during the same period. Additionally, the number of journals indexed in the Emerging Sources Citation Index (ESCI) experienced rapid growth, rising from 10 in 2011 to 21 in 2012.

Traditionally, the gerontology field has been at the forefront of research on aging in place or related concepts. However, since 2014, publications from the broader field of science have noticeably grown, becoming even more pronounced in 2018. Indeed, the trend of increased publications in the field of aging in place or related topics is reflected in the number of SCI index journals. The data show a significant increase over the years in the number of SCI index journals publishing research on this subject. In 2013, there were only 13 SCI index journals publishing relevant research. However, this number grew substantially in subsequent years, increasing to 26 in 2014, indicating a notable publication expansion for the field. In 2016, the number further increased to 37, indicating continued growth in research dissemination. However, there was a slight decrease in 2017, with 29 SCI index journals publishing research on aging in place or related topics.

The most significant surge occurred in 2018 when the number of SCI index journals publishing research on aging in place or related topics jumped to 54. This sharp increase in journals indicated the scientific research area’s growing recognition and interest in the field.

With the world’s population getting older, journals and research in this field experienced increasing trends that will continue in the future. Hence, this bibliometric analysis is expected to provide more insights into this domain’s research directions.

#### 3.1.2. Most Prolific Articles

Table 2 shows the most cited articles on aging in place (including similar concepts). Most of the studies on the list were published from 2008 to 2016. The most cited article (784 citations) on residential environments in aging in place in the WoS was “The meaning of ‘aging in place’ to older people,” written by Wiles et al. [5]. The study identified the practical meaning of aging in place for the elderly through focus group interviews, surveys, and conducting studies using sociological concepts like attachment to place. In second place was “Older adults’ reasons for using technology while aging in place”, written by Peek et al. [25] and cited 201 times. 

The difference between first and second place, where the first had more than three times more citations than the second, was the largest one observed, with the third-place paper having the same number of citations as the second-place one.

#### 3.1.3. Most Prolific Research Areas 

Studies on aging in place in the context of residential environments were performed in 116 diverse research areas. According to the WoS database, examples of these areas include gerontology, geriatric gerontology, public environmental occupational health, nursing, and urban studies. The field that stood out the most was, of course, gerontology. 

**Figure 2:**
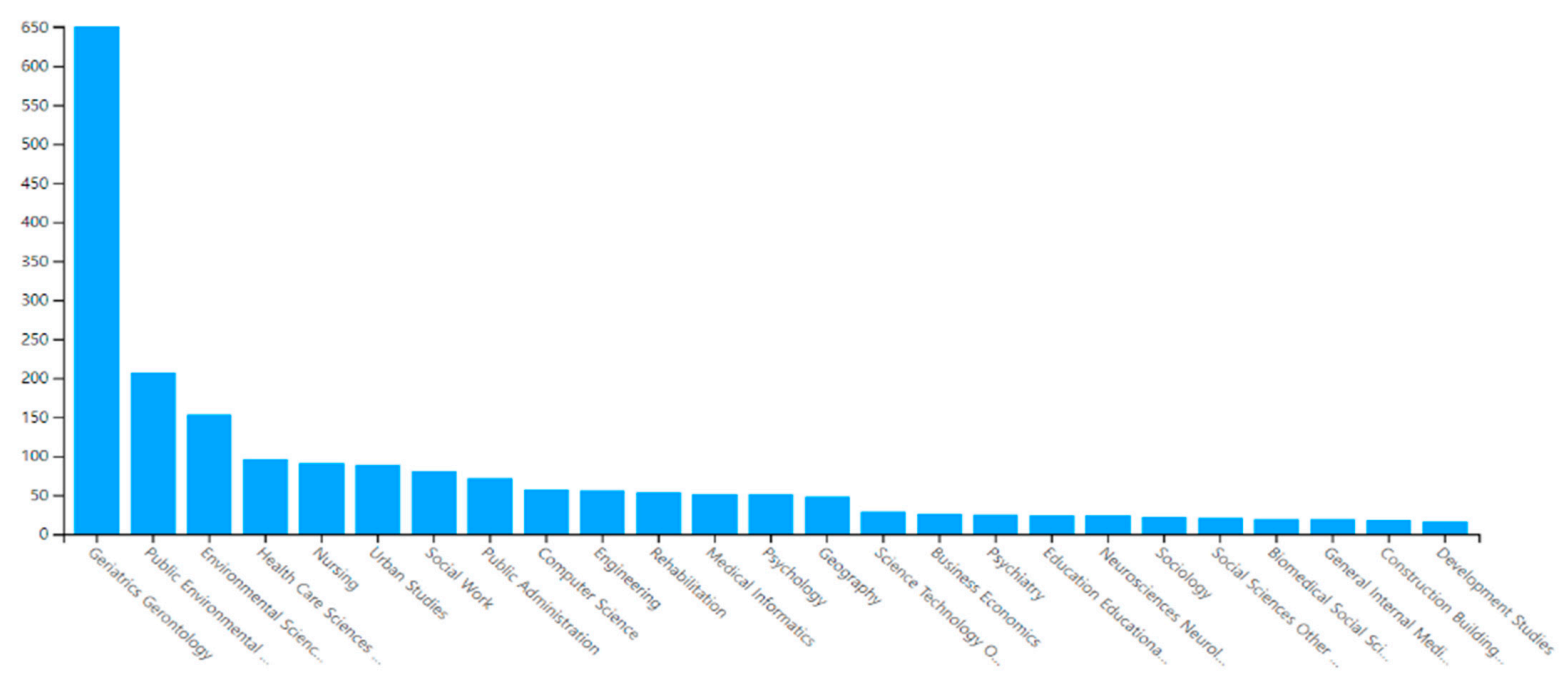
Research areas.

As shown in Figure 2 and Table 3, over 90% of the papers selected were conducted in the top seven areas. These research areas were classified as gerontology-related (gerontology and geriatric gerontology), health-related (public environmental occupational health, and nursing), environment-related (environmental sciences, environmental studies, and urban studies), and technology-related (computer science and engineering).

Gerontology is an established area that consistently leads the field. From 2004 to 2023, geriatric gerontology has appeared as a prolific field (Table 4). In 2004, technology-related research areas (computer science interdisciplinary application, computer science software engineering, computer science theory method, etc.) began to flow in, and currently, research fields such as health care sciences services, computer science information systems, and electrical engineering and electronics are producing results. The field of aging in place in a residential environment was grounded in qualitative research methods and case studies from the perspective of gerontology [29,34,35,36,37], but since 2004, technology-related fields, which can facilitate the implementation of residential environments for aging in place, increased [38,39] (Table 4).

#### 3.1.4. Most Prolific Authors

**Figure 3:**
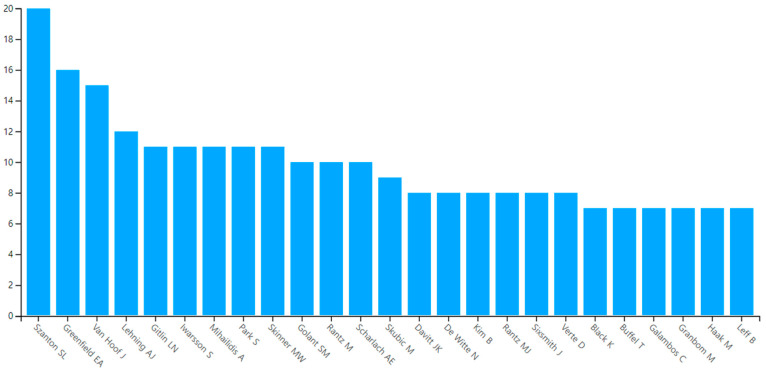
Most prolific authors.

In the residential environment field, the most productive 3 authors of research on aging in place among the 3443 authors in the WoS database were S.L. Szanton (20 publications), E.A. Greenfield (16 publications), and J. Van Hoof (14 publications). There was a 25% difference between the first-ranked and second-ranked authors (see Figure 3 and Table 5). The top 10 authors’ departments ranged from nursing, social work, health science, occupational science, and occupational therapy to geography. However, the top 10 authors’ regions did not vary, and most were from North America; six of them were from the United States, and two were from Canada. Other regions included Northern Europe (the Netherlands and Sweden) and the United Kingdom.

The exemplary work of S. L. Szanton, who is the most prolific author in this field, includes the paper titled “Home-based Care Program Reduces Disability and Promotes Aging in Place” (2016) which has received 119 citations, ranking 18th in citation ranking. Another notable publication is “Community Aging in Place, Advancing Better Living for Elders: A Bio-Behavioral-Environmental Intervention to Improve Function and Health-related Quality of Life in Disabled Older Adults” (2011), accumulating 115 citations and securing the 20th position in the ranking. Her main perspective is improving the quality of life for the elderly and achieving aging in place through mobility and home modification support. Her research has expanded from home-based care and home modification for the elderly in a community of people who have disabilities to successful implementation of aging in place through social participation support for the low-income group [40,41,42,43]. The productivity of the authors of the research papers selected for this study is shown in Table 5. 

#### 3.1.5. Most Prolific Affiliations and Countries

**Figure 4:**
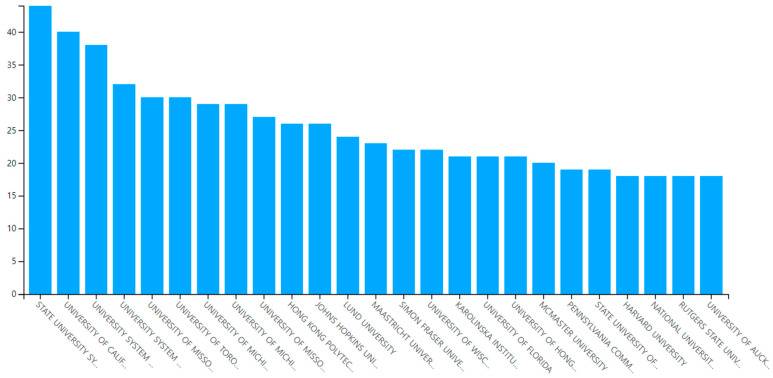
Prolific affiliations.

The selected papers were published by authors with 1539 affiliations across the world. The state university system of Florida (USA) was ranked first among the most prolific affiliations on aging in place in the context of a residential environment (see Table 6 and Figure 4). The top five most prolific affiliations in this research field were all located in the United States: the university systems of Florida, California, Maryland, Georgia, and Missouri. The most prolific country was the United States, where 40 percent (601) of the 1500 papers were produced. The most productive countries were the United States (40%), Canada (11.4%), and Australia (8.5%). 

### 3.2. Science Mapping

#### 3.2.1. Academic Collaboration Networks among Authors, Countries, and Organizations

(1) Authors

**Figure 5:**
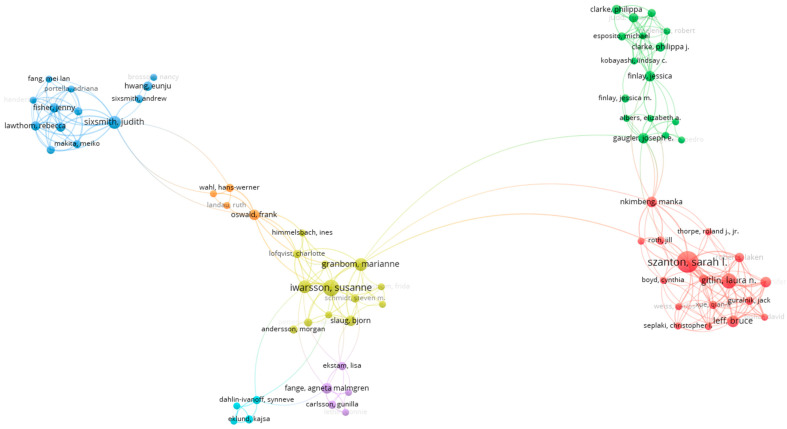
Mapping of authors’ collaboration networks.

The minimum number of documents per author was established as two for the bibliometric data. Out of 4514 authors, only 678 satisfied this condition to be included in the collaboration network map displayed in Figure 5. However, merely 72 out of these qualified to construct the detailed collaboration network map, also in Figure 5, where seven clusters consisting of 72 authors were discerned. The co-authorship analysis highlighted that prominent contributors like S.L. Szanton (who ranked first) and S. Iwarsson (who ranked sixth) have formed significant research collaborative bonds.

(2) Countries

**Figure 6:**
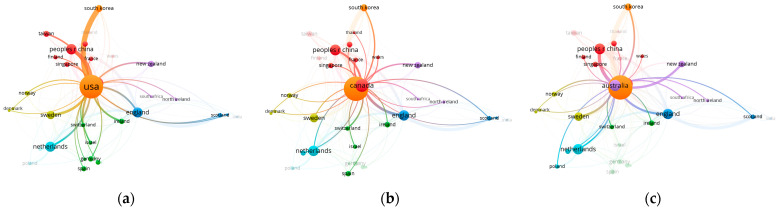
Co-authorship networks of countries: (**a**) US-centered; (**b**) Canada-centered; (**c**) Australia-centered.

The threshold for the bibliometric data was set as a minimum of five documents for a country. Of the 56 countries, 35 were selected. Figure 6 illustrates the distribution of studies across geographic regions. The circle sizes in the figure indicate the number of papers, and the collaboration strength is revealed by the distance and thickness of the links between circles of individual pairs. The prolific countries (the United States, Canada, and Australia) have well-established collaboration networks.

The United States occupies the most central position among all global collaborations, but, in particular, thick links reveal the strongest relationships with China and South Korea. Canada has a strong relationship with England, but China is also in its network. In its network, Australia has strong relationships with China, New Zealand, and England. As seen in Figure 6, Sweden and the Netherlands are relatively distant from the center, but they have numerous published papers. In contrast, Figure 6 shows that France, despite not having many published papers, is in close proximity to the United States and Canada, suggesting meaningful scholarly relationships.

(3) Organizations

The threshold in the bibliometric data for organizations was set as a minimum of five documents. Among the 1517 organizations, 144 were selected. 

**Figure 7:**
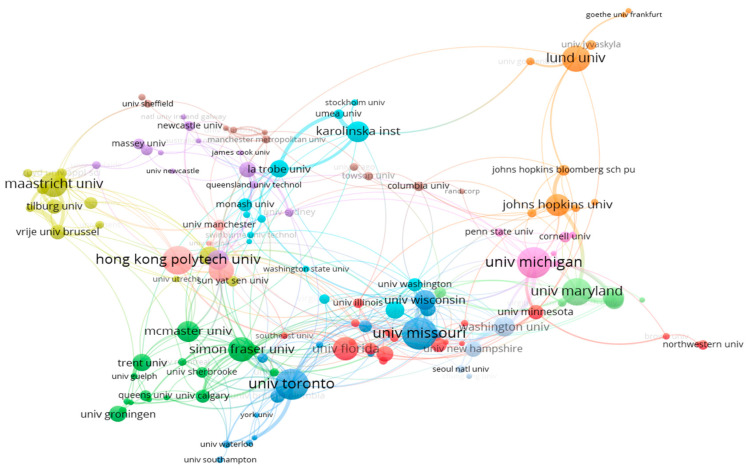
Co-authorship organization networks.

In Figure 7, a co-authorship analysis map displays relationships among 135 organizations that met the required criteria. Twelve distinct groups are apparent from the analysis. Prominent relationships are seen between several universities, including the University of Michigan, Lund University, the University of Missouri, the University of Toronto, Karolinska Institute, Johns Hopkins University, Hong Kong Polytechnic University, and Maastricht University. Among these connections, the University of Michigan’s partnerships with the University of Maryland and Johns Hopkins University emerged as the most robust.

#### 3.2.2. Major Research Area and Direction of the Research Domain

A co-occurrence analysis of items, or keywords, is utilized to reveal the logical structure of prevailing research. This analytical method denotes the frequency at which two items are found within the same record, reflecting their interrelation. Each cluster uncovered through this analysis can signify a principal area or direction in the ongoing research. This analysis was conducted by extracting items from the titles and abstracts of chosen publications and assessing how frequently they co-occur within the same document, thereby determining the correlation between the items. 

**Figure 8:**
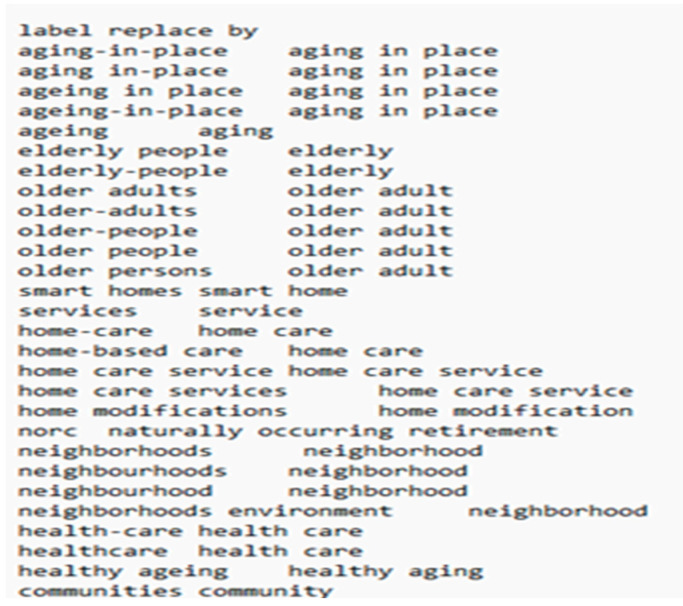
Thesaurus file.

The threshold of the text item occurrences was set as nine, defining the minimum frequency of a text item’s occurrence in a single document. Before performing this analysis, we merged different variations of keywords using the VOSviewer thesaurus file (Figure 8). Of the 3207 identified terms, 100 met the threshold; a relevance score was calculated for each of those that met the threshold.

**Figure 9:**
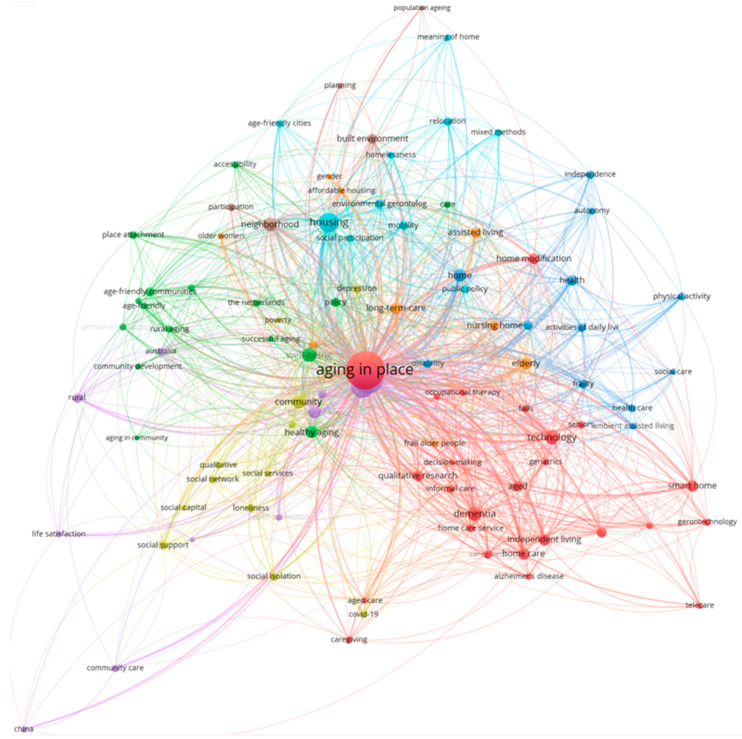
Co-occurrence network of author keywords (minimum of nine occurrences) in the WoS.

All terms were selected to create the co-occurrence map shown in Figure 9. The terms were divided into eight clusters: red (25 items), green (16 items), blue (12 items), yellow (12 items), purple (11 items), light blue (10 items), orange (9 items), and brown (5 items). Table 7 shows the top 10 most frequently co-occurring keywords and their total link strength colored according to cluster.

The dimension of each circle is proportional to the frequency of the depicted keyword. A larger circle implies a higher occurrence of the author keyword within the WoS databases. The distance between the elements of an individual pair demonstrates the subject similarity and its relative strength. Each circle color is assigned to keyword clusters of related topics. Figure 9 illustrates a network comprising eight distinct clusters, each representing a unique subfield within the research areas as identified in the WoS databases. The connections between specific keywords reflect the quantity of papers where those keywords appear together. 

Figure 9 shows that the core topics with the highest total link strength were “aging in place”, “older adult”, and “housing”. Eight subfields (clusters of author keywords) were identified in the research fields; these are shown in Table 8.

The findings reveal a wide range of co-occurring keywords in individual papers within the Web of Science database, highlighting the multidisciplinary and multifaceted nature of the field. In the keyword co-occurrence analysis, “aging in place”—the central focus of this study—showed the strongest association with topics like technology and smart homes, as seen in Cluster 1. This indicates that future research in this area is likely to be closely intertwined with technological advancements. Moreover, considerations of the physical environment intersected with spatial issues like home adaptations, enhancing mobility, the creation of age-friendly communities, and senior housing options. These considerations also have implications for public policy matters such as social engagement and homelessness, as demonstrated in Clusters 6 and 8. Recently, the COVID-19 pandemic has appeared in the aging in place field in areas related to social capital such as social networks, social services, and mental health (Cluster 4). Assisted living is being studied in relation to disabilities and long-term care and is particularly relevant to fields that require social roles, such as nursing homes and gender issues (Clusters 3 and 7).

#### 3.2.3. Relationships among the Leading Publications

**Figure 10:**
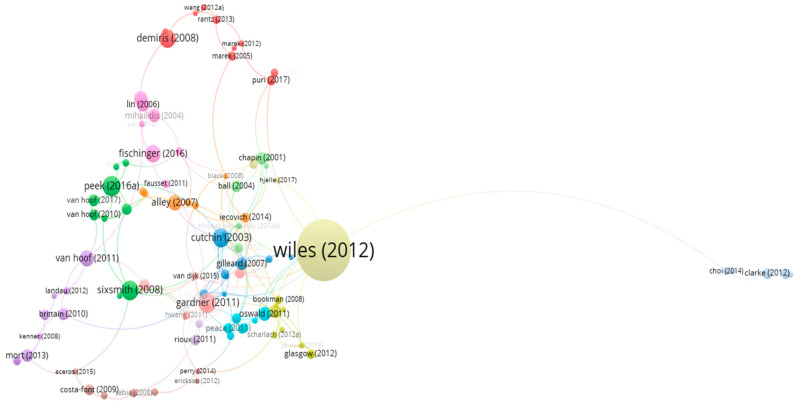
The citation analysis map of influential publications.

Citation analysis was utilized to discern the relationships between the principal publications in the field. The bibliometric data required a minimum of 40 citations per paper. Out of 1500 papers, 142 were shortlisted; however, only 100 papers satisfied the requirement to form the citation analysis map in Figure 10. In this refined selection, 14 clusters were recognized.

The map derived from the citation analysis denotes that the document by Wiles and Janine (2012) [5], being the most cited, retains its status as the most foundational publication. It is centrally located and maintains the most robust connections with other comparatively newer articles in the network map; it is closely connected to Oswald [44] in the same cluster and to 11 other clusters including Greenfield [45], Puri [46], Hjelle et al. [47], Means et al. [48], Van dijk et al. [49], Van hees [50], Skinner [51], Lager et al. [52], Granbom et al. [53], Hillcoat-Nallétamby [54], and Choi et al. [55] (see Figure 10). 

The second most cited document was “Older adults’ reasons for using technology while aging in place” by Peek et al. [25], which was linked to Luijkx [56], Peek [57], Van Hoof [33,58,59], Marston et al., and Cutchin [29]. The third strongest relationship in terms of number of links was “Natural neighborhood networks—Important social networks in the lives of older adults aging in place”, written by Gardner [26] (see Table 9). 

#### 3.2.4. Foundational Themes and Leading Publications 

Co-citation analysis is utilized to pinpoint foundational themes and predominant publications by examining the relationships among cited works. For the bibliometric data, a minimum threshold of 20 citations was established for each cited reference. Out of 49,278 cited references, 106 met the criteria and were chosen to formulate the co-citation map, subsequently organized into four distinct clusters. To address the limitations of co-citation analysis via VOSviewer, specifically with publications listed in reference format, a meticulous examination of each publication within the clusters was undertaken, focusing on total link strength, to isolate the foundational themes. The characteristics of thematic clusters were identified through a content analysis that examined the titles, keywords, and abstracts of the gathered papers.

**Figure 11:**
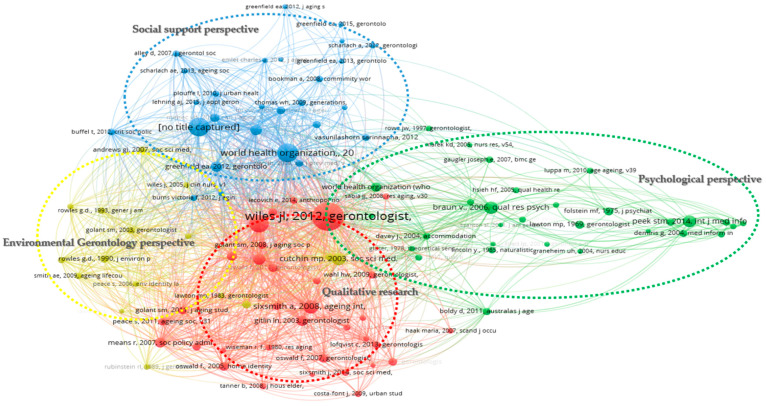
Co-citation analysis map showing foundational themes and seminal publications of clusters.

Among the 106 cited articles, four clusters were identified using the four broad perspectives in the research domain: (1) qualitative research—definition and related theory of AIP in red cluster 1: socio-physical environment and ecology theory of AIP; (2) psychological perspective—cognitive methodology in green cluster 2: cognition disorders, etiology, epistemology, and qualitative psychology; (3) social support perspective—community support and its measurable variables in blue cluster 3: social network, social services, care coordination and social ecology; and (4) environmental gerontology perspective: place integration, modification, optimization and place attachment—environment modification and its measurable variables in yellow cluster 4 (see Figure 11 and Table 10). The 20 influential publications in each cluster were identified.

#### 3.2.5. Bibliographic Coupling Analysis—The Development of Themes in the Literature

**Figure 12:**
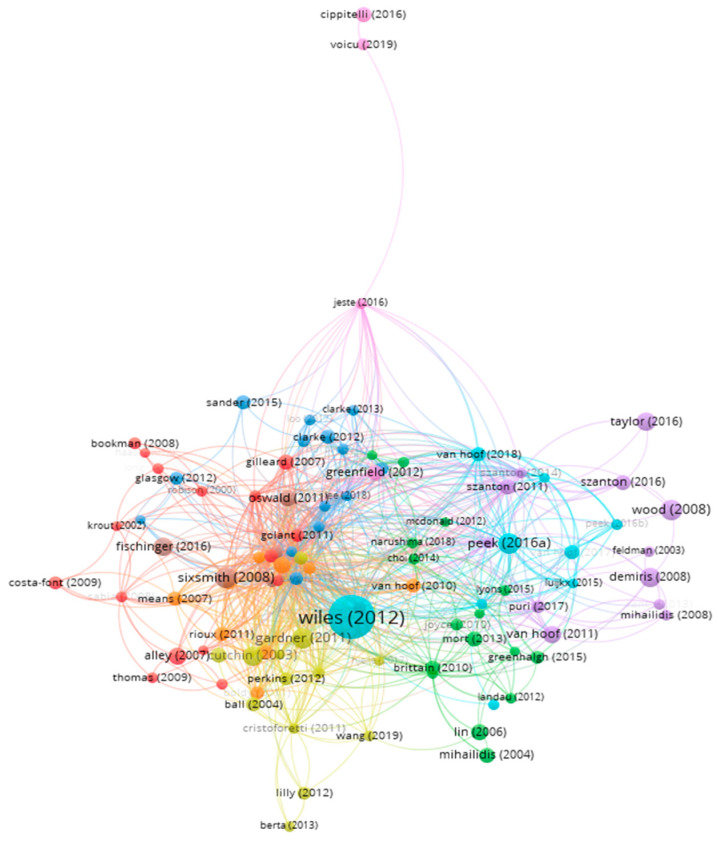
Bibliographic coupling analysis map showing development of literature themes and seminal publications in clusters.

Bibliographic coupling analysis was utilized to uncover evolving themes in literature by exploring the relationships between cited articles [28]. A threshold of a minimum of 50 citations per paper was established for the bibliometric data. From the initial 1500 papers, 104 were shortlisted, but only 99 met the criteria to form the bibliographic coupling analysis map depicted in Figure 12. These 99 papers were categorized into nine clusters. Contrary to the co-citation analysis map, the papers involved in the bibliographic coupling analysis are of a more recent publication date. (see Table 11).

Three or four papers were selected from the strongest total link for each cluster, and the characteristics of nine thematic clusters were identified by assessing the keywords, abstracts, and titles of the listed papers. The clusters are: Cluster 1 (red)—qualitative research from the epistemological perspective, such as questionnaires; Cluster 2 (green)—gerontechnology perspective related to home care/telecare; Cluster 3 (blue)—cognitive perspective related to social support; Cluster 4 (yellow)—environmental psychology related to geographical experience; Cluster 5 (purple)—home care/care models associated with health care; Cluster 6 (light blue)—gerontechnological perspective associated with acceptance and use of technology; Cluster 7 (orange)—environmental modifications related to optimization and independence; Cluster 8 (brown)—person–environment fit related to life satisfaction; and Cluster 9 (violet)—cognitive perspective.

## 4. Discussion

### 4.1. Implications of Findings for Aging in Place and the Residential Environment

#### 4.1.1. Expansion and Change in Leading Fields

The field of aging in place within a residential environment has experienced significant expansion and transformation in its key research domains. Initially centered around environmental gerontology, the field primarily focused on qualitative cognitive research and case studies and had a particular emphasis on understanding “place integration” and “sense of place attachment” among older adults [33,35,36,37,66,73,95]. 

In its early stages, gerontological research in aging in place emphasized the importance of aligning living environments with the individual needs, capabilities, and preferences of older adults [30,92]. Additionally, the notion of place attachment highlighted the positive emotional connection that older adults develop toward specific places [69]. Researchers in gerontology aimed to investigate the factors influenced the formation and maintenance of place attachment and explore ways to support strong emotional ties between older adults and their living environments [66,95].

The next notable area of growth in this field is in technology and information technology. Technological innovations such as artificial intelligence, the Internet of Things, smart devices, and health monitoring systems have played a pivotal role in enhancing aging in place environments and supporting older adults’ residential and well-being needs.

Since around 2004, there has been a significant increase in the number of research papers focusing on technology-related aspects in the context of aging in place. This surge in interest has been driven by recognizing technology’s potential to address the unique challenges and needs of older adults as they age in their own homes [38,39]. The intersection of technology and home care has particularly garnered substantial attention, expanding the field’s scope to include advancements in health care.

The integration of technology into aging in place has opened new possibilities for remote monitoring, telemedicine, smart home automation, and assistive technologies [33,96]. These technological advancements aim to support older adults in managing their health, maintaining independence, and improving their overall quality of life. Researchers and practitioners have increasingly recognized the importance of leveraging technology to provide personalized and efficient health care services, enabling older adults to age in place while receiving the necessary care and support.

Furthermore, research from disciplines like sociology, public administration, urban planning, medicine, and nursing has increasingly contributed to the field. As demographic shifts continue to change the composition of our society, particularly with an increase in the aged population, there is growing concern over housing policies that address the unique needs of seniors. These needs often include issues related to the ability for seniors to age in place and perform self-care tasks independently. As such, administrative research focusing on these aspects is on the rise [97,98,99,100]. Interest in the urban planning and architecture fields has been evident in the focus on creating age-friendly communities and implementing home modifications [86,87,88]. “Home modification” refers to adapting or altering residential environments to enhance older adults’ convenience and safety. In the fields of urban planning and architecture, there is growing interest in researching and implementing various modifications aimed at supporting older individuals in living independently. Technical collaboration is undertaken to implement spatial design, and this multidisciplinary collaboration involves improving accessibility, incorporating ergonomic principles, and implementing safety features and assistive technologies [76,85,96,101]. Public policies and public support for the socially underprivileged or elderly people with disabilities is predicted to develop in a close relationship with this field. The goal is to create comfortable and secure environments that enable older adults to maintain their independence within their own homes.

#### 4.1.2. Changing Perspectives on the Residential Environments of Those Aging in Place

The aging in place field has witnessed a significant shift in the perspective on residential environments, moving beyond an individual’s home itself to a broader view that encompasses the community and widening perspectives on psychological and social approaches from place attachment to the lens of well-being and support.

The initial focus in aging in place research revolved around the physical aspects of an individual’s home and modifications to support aging in place [43,87]. However, researchers and practitioners have recognized that residential environments extend beyond the confines of the home and encompass the surrounding community [10,12,49,92]. This expanded perspective acknowledges the importance of creating age-friendly communities that provide social connections, services, and opportunities for engagement. By considering the community an integral part of residential environments, aging in place initiatives aim to foster social inclusion, reduce isolation, and enhance older adults’ overall independent living.

Furthermore, there have been psychological and cognitive approaches, particularly the concept of place attachment, in understanding the significance of residential environments [34,36,69,95]. Place attachment refers to the emotional connection and sense of belonging that individuals develop toward their living spaces. Recognizing how place attachment impacts older adults’ well-being and satisfaction, researchers have explored ways to promote positive attachment and create supportive environments that enhance their quality of life [69].

In recent years, there has also been a shift toward viewing residential environments through the lens of well-being and support [69,102,103]. This broader perspective considers the physical, social, and psychological dimensions of living environments. It emphasizes the importance of creating environments that not only accommodate older adults’ physical needs but also foster their mental and emotional well-being. This includes considerations like access to health care services, social support networks, and opportunities for meaningful engagement and participation [52,104,105,106].

In summary, the evolving perspective of residential environments in the field of aging in place has encompassed a broader scope, recognizing the significance of the community, psychological approaches like place attachment, and the focus on well-being and support. By taking a holistic view, researchers and practitioners strive to create environments that promote older adults’ overall well-being, sense of belonging, and independence as they age in place. They emphasize the importance of exploring innovative approaches and methodologies for data collection and analysis in the context of aging in place.

### 4.2. Current View and Challenges for Future Research

As we look to the future, we anticipate that the accessibility of emerging big data sources will play a pivotal role in inspiring inventive strategies for acquiring residential environmental data related to aging in place. Utilizing these data resources can lead to a more comprehensive understanding of how the elderly interact with their residential environments and how they impact their well-being and overall quality of life.

**Figure 13:**
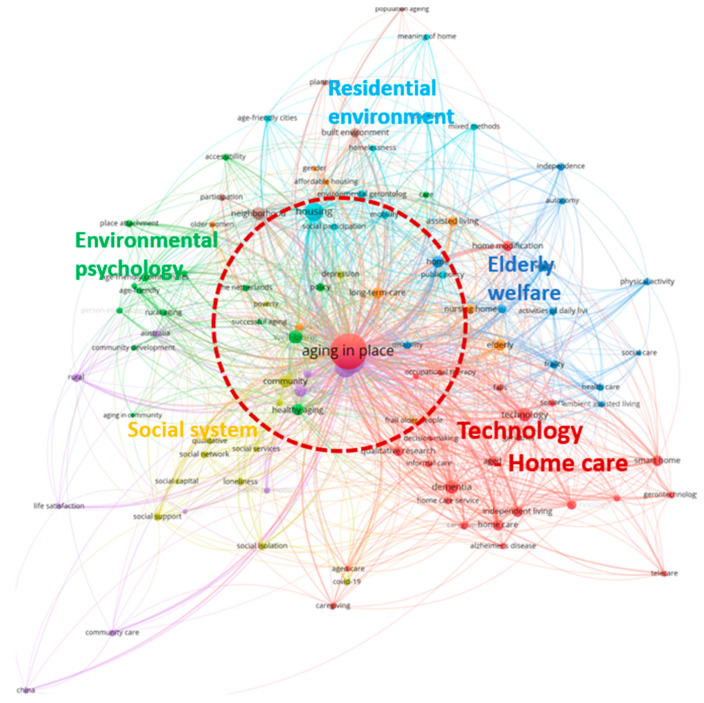
Present and future research topics.

The co-occurrence network map (Figure 13) further strengthened our findings by highlighting the central positioning of three critical terms—“housing”, “neighborhood”, and “community”—within the residential environment context. These terms are closely linked to the concept of “aging in place” and are integral to understanding the well-being of the elderly in their residential environments. The map’s central clustering of these terms, represented by distinct color clusters and large nodes, underscores their paramount importance in the co-occurrence analysis.

However, it is crucial to recognize that “aging in place” involves a multifaceted integration of various aspects. The co-occurrence analysis revealed eight thematic clusters that hold implications for researchers in this field. These clusters encompass crucial elements such as the residential environment, environmental psychology, social systems, technology/home care, individual elderly welfare, social support, and technology. Successful implementation of “aging in place” goes beyond focusing solely on the residential environment; instead, it necessitates the integration of these diverse aspects. Researchers must acknowledge the interconnectedness of these factors to develop effective strategies for supporting the elderly population in aging in place successfully.

By acknowledging the significance of these interconnected aspects and continuing to explore new methodologies and data sources, future researchers can drive meaningful advancements in the field of aging in place and improve the overall well-being and quality of life for the elderly.

## 5. Conclusions

This study conducted a comprehensive bibliometric analysis of aging in place within residential environments, offering a global perspective on publications spanning from 1991 to 2023. We successfully identified 1500 articles authored by 4365 individuals across 474 peer-reviewed journals utilizing keywords derived from an initial literature review focused on “aging in place” and residential environments.

While our study has made substantial contributions to this field, it is imperative to acknowledge the inherent limitations of the bibliometric analysis methodology employed. These limitations pertain to several key aspects: the restricted database scope limited to the Web of Science, the language constraint to English, a temporal focus spanning from 1991 to 2023, and a focus on peer-reviewed journals. In particular, our reliance solely on the Web of Science database as the primary data source introduced certain constraints. Despite its extensive coverage, the Web of Science may not encompass all academic disciplines, potentially omitting publications from specific research areas. Consequently, our analysis may not have fully captured the breadth of research related to aging in place.

Notwithstanding these limitations, our research has provided valuable insights into aging in place within residential environments, particularly within the confines of the Web of Science database. Future studies should consider a broader array of data sources to achieve a more comprehensive understanding of this dynamic research field.

In conclusion, this study establishes a foundational framework for researchers and practitioners in residential environment-related fields, facilitating a deeper comprehension of aging in place. It also issues a call to expand the scope of future investigations to encompass diverse data sources, further enriching our comprehension of this vital area.

## Figures and Tables

**Table 1:** Flowchart summarizing study process.

Step 1: Bibliometric technique and collecting data following PRISMA-ScR	2.1. Define the aim and scope of the research		
	RQ1. Which research area is leading in the field of aging in place in the context of residential environments? RQ2. What methodologies have been applied in aging in place research? RQ3. How are the theory and methodology of aging in place evolving? RQ4: What challenges and implications does aging in place have for residential environments?		
	2.2. Selection of Technique and Data		
	(1) Selection of databases with bibliometric data: the Web of Science (WoS)		
	(2) Selection of software tools for analysis: VOSviewer		
	(3) Identification: Selection of query wording and Boolean operators: Related to aging in place: (“aging in place” or “ageing in place”) or (“aging in community” or “ageing in community”) Related to residential environment: (housing or home or dwelling or residence or residential or community)	⇨	*n* = 1970
	(4) Screening: Selection of document types: peer-reviewed journals Excluded: grey literature, proceedings papers, review articles, meeting abstracts, book chapters, etc.	⇨	*n* = 1535
	(5) Selection of language: English	⇨	*n* = 1506
	(6) Eligibility: Selection of timespan (1991–2023), manually excluding review articles	⇨	*n* = 1500
Step 2: Bibliometric approach: performance analysis	3.1. Bibliometric approach/Performance analysis		
	(1) Descriptive bibliometric analysis of prolific research. Number of publications per year, total citations, and most productive authors and countries		
	(2) Descriptive bibliometric analysis of prolific articles		
	(3) Descriptive bibliometric analysis of prolific research areas		
	(4) Descriptive bibliometric analysis of prolific authors		
	(5) Descriptive bibliometric analysis of prolific affiliations and countries		
Step 3: Bibliometric analysis: science mapping	3.2. Science mapping		
	3.2.1. Co-authorship analysis of authors: Mapping the scientific collaboration of authors, countries, and organizations		
	3.2.2. Co-occurrence—keywords in WoS: Most frequently used words, author keywords, co-occurrence, and network of authors’ keywords		
	3.2.3. Citation analysis: Relationships among the leading publications		
	3.2.4. Co-citation analysis: Foundational themes and leading publications		
	3.2.5. Bibliographic coupling analysis: Development of themes in the literature		
Step 4:	4. Discussion		
	Conclusions and implications for future research		
	5. Conclusions		

**Table 2:** The top 10 most cited papers in the WoS search for residential environments in AIP.

	Author(s)	Year Published	Paper Title	Journal	Citation Count
1	Wiles, J.L.; Leibing, A.; Guberman, N.; Reeve, J.; Allen, R.E.S. [5]	2012	The meaning of aging in place to older people	*Gerontologist*	793
2	Peek, S.T.M.; Luijkx, K.G.; Rijnaard, M.D.; Nieboer, M.E.; van der Voort, C.S.; Aarts, S.; van Hoof, J.; Vrijhoef, H.J.M.; Wouters, E.J.M. [25]	2016	Older adults’ reasons for using technology while aging in place	*Gerontology*	201
3	Gardner, P. J. [26]	2011	Natural neighborhood networks—Important social networks in the lives of older adults aging in place	*Journal of aging studies*	201
4	Wood, A. D.; Sgankovic, J.A.; Virone, G.; Selavo, L.; He, Z.; Cao, Q.; Doan, T.; Wu, Y.; Fang, L.; Stoleru, R. [27]	2008	Context-aware wireless sensor networks for assisted living and residential monitoring	*Ieee network*	200
5	Sixsmith, A.; Sixsmith, J. [28]	2008	Ageing in place in the United Kingdom	*Ageing international*	187
6	Cutchin, M.P. [29]	2003	The process of mediated aging-in-place: a theoretically and empirically based mode l	*Social science & medicine*	183
7	Demiris, G.; Hensel, B.K.; Skubic, M.; Rantz, M. [30]	2008	Senior residents’ perceived need of and preferences for smart home sensor technologies	*International journal of technology assessment in health care*	173
8	Fischinger, D.; Einramhof, P.; Papoutsakis, K.; Wohlkinger, W.; Mayer, P.; Panek, P.; Hofmann, S.; Koertner, T.; Weiss, A.; Argyros, A.; Vincze, M. [31]	2016	Hobbit, a care robot supporting independent living at home: First prototype and lessons learned	*Robotics and autonomous systems*	167
9	Taylor, L.A.; Tan, A.X.; Coyle, C.E.; Ndumele, C.; Rogan, E.; Canavan, M.; Curry, L.A.; Bradley, E.H. [32]	2016	Leveraging the social determinants of health: What works?	*Gerontology*	165
10	Van Hoof, J.; Kort, H.S.M.; Rutten, P.G.S.; Duijnstee, M.S.H. [33]	2011	Ageing-in-place with the use of ambient intelligence technology: Perspectives of older users	*International journal of medical informatics*	145

**Table 3:** Top 10 most prolific research areas.

No.	Research Area	Record Count	% of 1133
1	Geriatric gerontology	578	38.533
2	Public environmental occupational health	238	15.867
3	Environmental sciences	207	13.8
4	Health care sciences services	91	6.067
5	Nursing	89	5.933
6	Urban studies	83	5.533
7	Social work	82	5.467
8	Public administration	81	5.4
9	Computer science	74	4.933
10	Engineering	69	4.6

**Table 4:** Most prolific categories during 2004~2012.

No.	2003	2004	2006	2008	2010
1	Public environmental occupational health	Gerontology	Geriatric gerontology	Gerontology	Gerontology
2	Gerontology	Geriatric gerontology	Gerontology	Geriatrics gerontology	Geriatrics gerontology
3	Family studies	Nursing	Medical information	Health care sciences services	Public environmental occupational health
4	Nursing	Architecture	Business finance	Medical informatics	Biomedical social sciences
5	Psychology (developmental)	Computer science hardware architecture	Computer science information systems	Nursing	Computer science interdisciplinary applications
6	Psychology (multidisciplinary)	Computer science information systems	Computer science Interdisciplinary applications	Regional urban planning	Health policy services
7	Social work	Computer science software engineering	Computer science software engineering	Rehabilitation	Rehabilitation
8		Mathematical computational biology	Computer science theory method	Computer science hardware architecture	Environmental sciences ecology
9		Medical informatics	Health care science	Computer science information systems	Health care sciences services
10		Psychology	Mathematical computational biology	Electrical engineering and electronics	Engineering

**Table 5:** The top 10 most prolific authors.

	Author	Institution	Country	Documents
1	Szanton, S.L.	Johns Hopkins University School of Nursing	USA	20
2	Greenfield, E.A.	Rutgers State University School of Social Work	USA	16
3	Van Hoof, J.	Hague University of Applied Sciences Dept. of Social Work & Education	The Netherlands	15
4	Lehning, A.J.	University of Maryland School of Social Work	USA	12
5	Gitlin, L.N.	Drexel University School of Nursing	USA	11
6	Iwarsson, S.	Lund University Dept. of Health Sciences	Sweden	11
7	Mihailidis, A.	University of Toronto Department of Occupational Science & Occupational Therapy	Canada	11
8	Park, S.	Washington University Institute for Public Health	USA	11
9	Skinner, M.W.	Trent University Dept. of Geography	Canada	11
10	Golant, S.M.	University of Florida Dept. of Geography	USA	10

**Table 6:** The top 10 most prolific affiliations and countries.

	Publication Titles	Record Count	Country	Record Count
1	University of Florida system	44	USA	601
2	University of California system	40	Canada	171
3	University of Maryland system	38	Australia	128
4	University of Georgia system	32	China	114
5	University of Missouri system	30	England	106
6	University of Toronto	30	The Netherlands	106
7	University of Michigan	29	Sweden	74
8	University of Michigan system	29	South Korea	51
9	University of Missouri-Columbia	27	New Zealand	41
10	Hong Kong Polytechnic University	26	Taiwan	38

**Table 7:** Top 10 most frequently co-occurring keywords and their relationships colored according to cluster.

	Keyword	Occurrences	Total Link Strength
1	Aging in place	634	972
2	Older adult	300	538
3	Housing	90	311
4	Well-being	49	119
5	Technology	43	117
6	Dementia	55	113
7	Community	45	111
8	Neighborhood	43	110
9	Home	42	93
10	Quality of life	39	87

**Table 8:** Keywords according to cluster.

Cluster 1 (Red)	Cluster 2 (Green)	Cluster 3 (Blue)	Cluster 4 (Yellow)
Aging in place Assistive technology Gerontechnology Independent living Smart home Dementia Technology acceptance	Age-friendly community Well-being Community development Place attachment Senior housing Healthy aging Policy	Activities of daily living AAL (ambient assisted living) Disability Frailty Health care Independence Social care	Community COVID-19 Social capital Social network Social isolation Mental health Social service Social support Loneliness
**Cluster 5 (Purple)**	**Cluster 6 (Light Blue)**	**Cluster 7 (Orange)**	**Cluster 8 (Brown)**
Community care China Australia Rural Quality of life	Environmental gerontology Mobility Relocation Social participation Homeless Public policy	Assisted living Frail older people Long term care Nursing home Gender	Built environment. Home modification Neighborhood Planning

**Table 9:** Top three most influential publications with the strongest relationships based on citation analysis.

	Author(s)	Year Published	Paper Title	Citation Count	Links
1	Wiles [5]	2012	The meaning of “aging in place” to older people	793	12
2	Peek [25]	2016 a	Older adults’ reasons for using technology while aging in place	201	6
3	Gardner [26]	2011	Natural neighborhood networks—Important social networks in the lives of older adults aging in place	201	3

**Table 10:** Top 12 most co-cited publications identified through co-citation analysis of cited references showing thematic clusters and seminal publications ranked according to link strength.

Foundational Theme	Seminal Publication	Topics/Keywords	Citations	Total Link Strength
Cluster 1 (red) of 33 documents: Qualitative research— definition and theory of AIP	Wiles et al. [5]	Aging in place (AIP) Home and community-based care Interview	270	1290
	Oswald et al. [44]	Housing/community/neighborhood Life satisfaction Socio-physical environment Questionnaire	45	298
	Sixsmith [28]	Aging in place (AIP) Telecare Questionnaire	88	530
	Means [48]	Aging in place Homelessness Vulnerable housing situations	58	398
	Wahl [60]	Ecology theory of AIP Physical–spatial–technical environment Person–environment resources	66	482
Cluster 2 (green) of 30 documents: Psychological perspective—cognitive methodology	Peek et al. [25]	Independent living Assist technology e-health	74	255
	Folstein et al. [61]	Cognition disorders Etiology	43	113
	Braun et al. [62]	Epistemology Qualitative psychology	77	288
	Gitlin et al. [63]	Home care Rehabilitation Disability/frailty	29	82
Cluster 3 (blue) of 27 documents: Social support perspective—community support and its measurable variables	World Health Organization [1]	Global age-friendly cities	52	213
	Gardner [26]	Communities Social network Natural neighborhood network	62	415
	Greenfield [45]	Social services Care coordination Community interventions Community partnerships	59	434
	Menec et al. [64]	Social environment Physical environment Community environment Healthy aging Social ecology	44	297
	Lui [65]	Age-friendly community Planning and governance Aging policy	41	276
Cluster 4 (yellow) of 16 documents: Environmental gerontology perspective— Environment modification and its measurable variables	Cutchin [29]	Aging-in-place Place integration Assisted living residences	79	493
	Golant [66]	Place attachment Environmental behaviors	33	246
	Wahl et al. [67]	Nursing home Modification/optimization Socio-physical environment	25	191
	Rowels [68]	Personal identity Autobiographical insideness	41	359
	Gilleard [69]	Aging in place Place attachment CASP 19	40	300

**Table 11:** Seminal publications and nine thematic clusters using bibliographic coupling analysis of cited references.

Foundational Theme	Seminal Publication	Title	Citations	Total Link Strength
Cluster 1 (red) of 16 documents: Qualitative research— epistemological perspective	Iecovich [70]	Services for the elderly population in Israel: the need for a national master plan	83	97
	Buffel [71]	Theorizing the relationship between older people and their immediate social living environment	58	74
	Golant [72]	The quest for residential normalcy by older adults: Relocation but one pathway	91	58
	Sabia [73]	There’s no place like home: A hazard model analysis of aging in place among older homeowners in the PSID	66	21
Cluster 2 (green) of 16 documents: Gerontechnology perspective—home care/telecare	Brittain [74]	Ageing in place and technologies of place: The lived experience of people with dementia in changing social, physical, and technological environments	105	30
	Piau [75]	Aging society and gerontechnology: A solution for an independent living?	50	29
	Mort [76]	Ageing with telecare: Care or coercion in austerity?	111	9
Cluster 3 (blue) of 14 documents: Cognitive perspective—social support	Andrews [77]	Re-spacing and re-placing gerontology: Relationality and affect	80	137
	Van dijk [49]	The ideal neighborhood for ageing in place as perceived by frail and non-frail community-dwelling older people	57	79
	Clarke [78]	Cognitive decline and the neighborhood environment	89	34
	Lee [79]	Cognition in context: The role of objective and subjective measures of neighborhood and household in cognitive functioning in later life	50	32
Cluster 4 (yellow) of 13 documents: Environmental psychology—geographical experience	Peace [80]	‘Option recognition’ in later life: variations in ageing in place	89	88
	Cutchin [29]	The process of mediated aging-in-place: a theoretically and empirically based model	33	246
	Löfqvist [81]	Voices on relocation and aging in place in very old age—A complex and ambivalent matter	78	53
	Gardner [26]	Natural neighborhood networks — Important social networks in the lives of older adults aging in place	201	40
	Cristoforetti [82]	Home sweet home: The emotional construction of places	65	37
Cluster 5 (purple) of 12 documents: Home care/care model—health care	Szanton [41]	CAPABLE trial: A randomized controlled trial of nurse, occupational therapist and handyman to reduce disability among older adults: Rationale and design	79	493
	Szanton [40]	Community aging in place, advancing better living for elders: A bio-behavioral-environmental intervention to improve function and health-related quality of life in disabled older adults	115	52
	Fausset [83]	Challenges to aging in place: Understanding home maintenance difficulties	64	44
	Puri [46]	User acceptance of wrist-worn activity trackers among community-dwelling older adults: Mixed method study	80	33
Cluster 6 (light blue) of 9 documents: Gerontechnological perspective— acceptance and use of technology	Van Hoof [59]	The challenges of urban ageing: Making cities age-friendly in Europe	95	82
	Peek [25]	Older adults’ reasons for using technology while aging in place	201	72
	Marston [84]	“Who doesn’t think about technology when designing urban environments for older people?” A case study approach to a proposed extension of the WHO’s age-friendly cities model	51	60
	Golant [85]	A theoretical model to explain the smart technology adoption behaviors of elder consumers (Elderadopt)	50	57
Cluster 7 (orange) of 9 documents: Environmental modification	Phillips [86]	Older people and outdoor environments: Pedestrian anxieties and barriers in the use of familiar and unfamiliar spaces	68	77
	Hwang [87]	Impacts of home modifications on aging-in-place	65	64
	Hillcoat-Nallétamby [88]	Moving beyond ‘ageing in place’: older people’s dislikes about their home and neighbourhood environments as a motive for wishing to move	70	61
	Tanner [43]	Restoring and sustaining home: The impact of home modifications on the meaning of home for older people	99	48
Cluster 8 (brown) of 6 documents: Person–environment fit—life satisfaction	Nygren [89]	Relationships between objective and perceived housing in very old age	54	99
	Oswald et al. [44]	Is aging in place a resource for or risk to life satisfaction?	125	75
	Fänge [90]	The home is the hub of health in very old age: Findings from the ENABLE-AGE Project	71	72
	Stones [91]	‘At home it’s just so much easier to be yourself’: Older adults’ perceptions of ageing in place	83	70
	Sixsmith [28]	Ageing in place in the United Kingdom	187	30
Cluster 9 (violet) of 4 documents: Cognitive perspective	Jeste [92]	Age-Friendly Communities Initiative: Public health approach to promoting successful aging	58	39
	Greenfield [45]	Using ecological frameworks to advance a field of research, practice, and policy on aging-in-place initiatives	118	30
	Voicu [93]	Human physical activity recognition using smartphone sensors	72	3
	Cippitelli [94]	A human activity recognition system using skeleton data from RGBD sensors	113	2

## Data Availability

The data used in this study are openly available in the Web of Science Core Collection.
